# The reciprocal changes in dominant species with complete metabolic functions explain the decoupling phenomenon of microbial taxonomic and functional composition in a grassland

**DOI:** 10.3389/fmicb.2023.1113157

**Published:** 2023-03-16

**Authors:** Huaiqiang Liu, Frank Yonghong Li, Jiayue Liu, Chunjun Shi, Kuanyan Tang, Qianhui Yang, Yu Liu, Qiang Fu, Xiaotian Gao, Ning Wang, Wei Guo

**Affiliations:** ^1^Ministry of Education Key Laboratory of Ecology and Resource Use on the Mongolian Plateau and Inner Mongolia Key Laboratory of Grassland Ecology, School of Ecology and Environment, Inner Mongolia University, Hohhot, China; ^2^Collaborative Innovation Center for Grassland Ecological Security, Ministry of Education of China, Hohhot, China

**Keywords:** grazing, phosphorus addition, functional redundancy, bistability, broad/specialized metabolic functions, grassland

## Abstract

The decoupling of microbial functional and taxonomic components refers to the phenomenon that a drastic change in microbial taxonomic composition leads to no or only a gentle change in functional composition. Although many studies have identified this phenomenon, the mechanisms underlying it are still unclear. Here we demonstrate, using metagenomics data from a steppe grassland soil under different grazing and phosphorus addition treatments, that there is no “decoupling” in the variation of taxonomic and metabolic functional composition of the microbial community within functional groups at species level. In contrast, the high consistency and complementarity between the abundance and functional gene diversity of two dominant species made metabolic functions unaffected by grazing and phosphorus addition. This complementarity between the two dominant species shapes a bistability pattern that differs from functional redundancy in that only two species cannot form observable redundancy in a large microbial community. In other words, the “monopoly” of metabolic functions by the two most abundant species leads to the disappearance of functional redundancy. Our findings imply that for soil microbial communities, the impact of species identity on metabolic functions is much greater than that of species diversity, and it is more important to monitor the dynamics of key dominant microorganisms for accurately predicting the changes in the metabolic functions of the ecosystems.

## 1. Introduction

The global imbalance of nitrogen (N) and phosphorus (P) may cause significant changes in organisms and ecosystems and cause future P limitations in natural ecosystems (Peñuelas et al., 2013). P addition affects the ecosystem mainly by changing the stoichiometry of N and P in its components (Peñuelas et al., 2013). However, its effects on microbial and plant communities depend on soil nutrient constraints (Avolio et al., 2014; Leff et al., 2015).

Even though the role of microorganisms has been recognized as the basis for plant species coexistence and positive diversity-productivity relationships (Klironomos, 2002; Mangan et al., 2010; Kulmatiski et al., 2012), how fertilization drives the changes in microbial community composition remains uncertain (Widdig et al., 2020; Lekberg et al., 2021). The prevailing view is that application of P mainly regulates the composition of soil microbial communities by altering soil pH (Fierer and Jackson, 2006; Fierer et al., 2009; Guo et al., 2010). Soil pH also affects microbial functions by affecting functional genes such as phosphatase (the enzyme that hydrolyzes organic P into orthophosphate, which is the only biologically available form of P for plants and microorganisms in soil) genes (Ragot et al., 2016). Long-term P input increases microbial P fixation by decreasing the relative abundance of *phoR* and increasing the relative abundances of *pit*, but does not alter the abundance of most *Pho* regulation genes that act as biological markers (Dai et al., 2019).

Human activities may affect the nutrient cycling of grassland ecosystems by direct input of nutrients, or by grazing and mowing on the grasslands (Yang et al., 2012). The effects of grazing on the grassland ecosystem fall into three areas: vegetation removal, fecal deposition, and trampling (Yang et al., 2012). Vegetation removal and fecal deposition accelerate the carbon (C) and N cycles, respectively (Guitian and Bardgett, 2000; Kohler et al., 2005), and the direction of their net effect depends on environmental conditions (Wardle, 2004) and grazing management. Trampling directly results in soil compaction, which leads to less pore space, less permeability, and less water availability (Byrnes et al., 2018). Through the above three aspects, grazing can significantly alter the response ratio of microbial genes related to C and N cycle in the soil, for example, decrease the abundance of *gdh* gene but increase that of *ureC* gene, thus affect the C and N related functions of soil microorganisms (Yang et al., 2012).

Microorganisms are the main regulators of the nutrient cycle of ecosystems, and the direct or indirect human impacts on the nutrient cycle alter the fate of microorganisms and ecosystems (Widdig et al., 2020). Previous studies have shown that grazing and P addition alter microbial community composition and functions associated with metabolic cycles to some extent, but tend to focus on a small number of differentially abundant genes. On the other hand, the existence at very small spatial scales of an extremely rich microbial community with both non-random and complex networks and structures has led to the hypothesis that a highly diverse microbiome is functionally redundant (Allison and Martiny, 2008). This hypothesis is derived from plant ecology that higher biodiversity supports higher functional redundancy and functional diversity (Hector and Bagchi, 2007; Isbell et al., 2011; Huang et al., 2018). However, in the study of microbial ecology, the introduction of the above macro-ecological theory does not play the expected effect.

For example, it has been reported that functional gene abundance has no relationship with the structure of microbial community in different seasons (Graham et al., 2014), under different land use patterns (Attard et al., 2010) or at different geographical distances (Louca et al., 2016a), and even has a very small range of change (Attard et al., 2010; Graham et al., 2014; Louca et al., 2016a). A large number of studies have found that the taxonomic composition within microbial functional groups seems to be shaped by factors other than those to form the functional structure of the community, that is, the taxonomic and functional components of microorganisms are “decoupled” (Fernández et al., 1999; Louca et al., 2016a, 2016b). Low genetic linkage of intra-genomic traits caused by horizontal gene transfer in microorganisms (Hehemann et al., 2016) and the role of convergent evolution (David and Alm, 2010) together lead to impaired phylogenetic signals of many traits, which is thought to be the cause of the “decoupling” of taxonomic and functional components of microorganisms (Louca et al., 2016b), but in fact, the concrete manifestation of this “decoupling” is only the invariance of the abundance of functional genes. In addition, ecosystem functions mainly involve microbial metabolic functions, most of which are related to more complex functional traits and the interaction of multiple parts of the genome, including epistasis among genes, which increases the difficulty in understanding the relationship between microbial biodiversity and function (Martiny et al., 2015).

In spite that the “decoupling” occurs in many of the above mentioned studies, we have not found a field study of the “decoupling” between taxonomic composition and metabolic functions associated with nutrient cycling by direct and indirect changes in nutrients. Previous microcosm study of bistability mechanisms in microbial communities has found that antibiotic inhibition favors high-abundance strains against the invasion of other strains, while low-abundance strains are unable to invade other strains (Wright and Vetsigian, 2016). Combined with the high metabolic versatility of actinomycetes (Van Bergeijk et al., 2020), we hypothesize that the relative abundance and metabolic gene abundance of a few key actinomyces dominate the metabolic functions of the entire microbial community, which cause the “decoupling” of taxonomic and functional components. If this is true, then the effects of oligotrophic or eutrophic soils on antibiotic production and growth of key actinomycetes would control ecosystem functioning.

Here, taking the soil samples from a field experiment studying the effects of grazing and P addition on grassland ecosystems, we studied the effects of grazing and P addition on the taxonomic and functional composition of soil microbial communities at species level using the metagenomics. We focused on the soil microbiome of grassland bulk soil, which to some extent disentangled the influence of plant rhizosphere host-related environments on soil microbial communities (Liu et al., 2023).

## 2. Materials and methods

The field study was conducted in the Xilingol Grassland Demonstration Pasture of Inner Mongolia University, located in Xilinhot City, Inner Mongolia Autonomous Region, China (116°31′E, 44°15′N, elevation 1,146 m). The area has a temperate semi-arid steppe climate. Mean annual temperature is about 2.6°C, with monthly mean temperature lowest in January (−23.5°C) and highest in July (25.2°C). Mean annual precipitation is about 315 mm, falling mainly during the period from June to September. The soil is chestnut soil. The grassland was dominated by *Stipa grandis*, *Leymus chinensis*, and *Cleistogenes squarrosa*. For a more detailed description of the studied ecosystem, see Liu et al. (2023).

The experimental grassland had been a natural steppe grazing land before 2012 when grazing was banned for grassland recovery. The experimental grassland plots were set up in 2016, and started the grazing treatments in June 2017. Each plot area is 0.25 hm^2^ (50 m × 50 m), and the full experiment had four grazing intensity treatments, and each treatments is replicated by 4 plots. Non-grazing (G–) and heavy grazing treatment plots (G+) were used in this study, and the G+ plots were grazed by a herd of 9 Mongolian sheep (2-year-old, with an average body weight of 33 kg) three times in June, July, and August each year. In August 2019, two areas of 4 m^2^ (2 m × 2 m) were set out for P addition treatment in each G– and G+ plots. One area was applied with 40 g superphosphate (containing 16% P_2_O_5_) within 20 l water (P+), and the other area was applied 20 l water only (P–). The P addition was done once every 14 days starting in June and ending in mid-August from 2019 to 2021, and the dose of P treatment (40 g superphosphate) is in harmony with local environmental conditions. That is, the experiment in the present study consists of four treatments (G–P–, G–P+, G + P–, and G + P+) in a random block design.

### 2.1. Soil sampling

Soil samples were collected in August 2021. Five soil samples were collected using a 3.8 cm diameter drill at each treatment area of 4 m^2^ using five quadrats of 0.5 m × 0.5 m placed along the diagonal line, mixed evenly and put in plastic bags (remove rocks, roots, litter, etc.), put into an incubator with ice cubes, and transferred back to the lab as soon as possible. The 16 soil samples were screened by 2 mm and stored in the refrigerator at −80°C for subsequent microbial metagenomics sequencing.

### 2.2. DNA extraction and metagenomic sequencing

Utilize E.Z.N.A.® Soil DNA Kit (Omega Bio-Tek, Norcross, GA, United States) for sample DNA extraction. After genomic DNA extraction, TBS-380 was used to detect the concentration of DNA, NanoDrop2000 to detect DNA purity, and 1% agarose gel to determine the extract quality of the DNA. The DNA was fragmented by Covaris M220 (Gene Company Limited, China), screened for about 400 bp of fragments, and finally used NEXTflexTM Rapid DNA-Seq (Bioo Scientific, Austin, TX, USA) to construct a Paired-end library. Adapters containing the full complement of sequencing primer hybridization sites were ligated to the blunt-end of fragments. Paired-end sequencing was performed on Illumina NovaSeq (Illumina Inc., San Diego, CA, United States) at Majorbio Bio-Pharm Technology Co., Ltd. (Shanghai, China) according to the manufacturer’s instructions^1^. Sequence data associated with this paper have been deposited in the NCBI Sequence Read Archive under BioProject accession number PRJNA876377.

### 2.3. Sequence quality control and genome assembly

The raw reads of metagenomic sequencing were taken and the adaptor sequences were removed by Fastp (Chen et al., 2018)^2^ (version 0.20.0), and the low-quality reads were trimmed and removed (reads with N bases, a minimum length threshold of 50 bp and a minimum quality threshold of 20) to generate clean reads. Assemble these high-quality reads into contigs using MEGAHIT (Li et al., 2015) (parameters: kmer_min = 47, kmer_max = 97, step = 10)^3^ (version 1.1.2), and the contigs over 300 bp were selected as the final assembling result. Identifying Open reading frames (ORFs) in contigs with MetaGene (Noguchi et al., 2006)^4^. Select the ORFs with length of ≥100 bp and translate it into amino acid sequences using the NCBI translation table, then use CD-HIT (Fu et al., 2012)^5^ (version 4.6.1) to construct (90% sequence identity, 90% coverage) a non-redundant gene catalog. Finally take advantage of SOAPalligner (Li et al., 2008)^6^ (version 2.21) mapped the quality controlled reads to the non-redundant gene catalog with 95% identity and evaluated the gene abundance of each sample. Gene abundance was calculated by RPKM (Reads Per Kilobase Million), that is, the number of reads per one million sequences per gene in a unit of one thousand bases.

### 2.4. Annotation and screening of species and functions

Representative sequences of non-redundant gene catalog were annotated based on the NCBI NR database using blastp as implemented in DIAMOND v0.9.19 with e-value cutoff of 1e-5 using Diamond (Buchfink et al., 2014)^7^ (version 0.8.35) for taxonomic annotations. The KEGG annotation was conducted using Diamond (Buchfink et al., 2014) against the Kyoto Encyclopedia of Genes and Genomes database^8^ (version 94.2) with e-value cutoff of 1e-5.

While next-generation sequencing allows the capture of vast microbial diversity, much of that diversity has little effect on the direction of research. For example, the inclusion of a large number of microorganisms that do not play an important role in ecosystem functioning would result in noise and blur the true diversity-interaction or diversity-function relationships (Wagg et al., 2019). For these reasons, some ecologists have called for the use of microbial functions rather than taxonomic characteristics to distinguish microbial niches at environmental gradients (Green et al., 2008). So, using taxonomic composition within functional groups, we investigated the community composition and interaction networks of species that perform similar functions but have distant ties of relatedness (Fernández et al., 1999; Louca et al., 2016a; Chen et al., 2022).

Broad (“broad”) metabolic functions are the basic metabolic function of microorganisms, which exist in all cells (Crowther et al., 2019). Specialized (“narrow”) metabolic functions exist only in taxa that partially perform specific metabolic functions (Schimel and Gulledge, 1998). These specialized metabolic functions are thought to have less functional redundancy and a more pronounced microbial biodiversity-ecosystem function (BEF) relationship (Hall et al., 2018). A comparative study of global metagenomic data has shown that although the degree of redundancy in “narrow” functions is slightly lower than that in “broad” functions, there is still a high degree of functional redundancy in the “narrow” functions (Chen et al., 2021, 2022). This may indicate that both of them have functional complementarity. Despite the uncertainty, we refer to and slightly modify the classification of the broad and specialized metabolic functions based on the KEGG database by Xun et al. (2021), for a tentative exploration (Supplementary Table S1). The functions related to N cycle are mainly in the specialized metabolic functions, while those related to C and P cycles are mainly in the broad (Xun et al., 2021).

### 2.5. Statistical analysis

We used iPath3^9^ to visualize the KEGG metabolic pathway (Darzi et al., 2018). To discover the potential interactions of functional genes in microbial communities under different treatments, Molecular Ecological Network Analysis Pipeline (Deng et al., 2012)^10^ was used to realize microbial functional networks. MENA applies random matrix theory (RMT) to microbiome data and attempts to be robust to noise and arbitrary thresholds (Weiss et al., 2016). The converted data matrix was uploaded with default settings to construct the network, setting the parameter “calculation order” to “decrease the cutoff from top,” setting the parameter “scan speed” to “regress Poisson distribution only,” and setting the RMT threshold as high and as close as possible to allow comparison between networks. Based on these conditions, the overall properties of the network based on the default settings were analyzed using “greedy modularity optimization,” and the networks were finally visualized using the Cytoscape (Shannon, 2003). Several network indices were used to characterize the network, including (1) the average connectivity (avgK) describing the complexity of the network; (2) the average clustering coefficient (avgCC) measuring the extent of modular structures in the network; (3) the average path distance (GD) indicating the degree of dispersion of nodes in the network; (4) the geodesic efficiency (E) representing the closeness of all nodes; and (5) the harmonic geodesic distance (HD), which was the reciprocal of E, and similar to GD, but was more suitable for messy networks. The redundancy of alternative paths was measured by natural connectivity, e.g., the average eigenvalue changes monotonically with the removal or addition of edges, using the spectral measure of stability of complex networks (Wu et al., 2010). Although there was no clear threshold value, if the natural connectivity decreased gently first and then steeply with the increase of the number of removed nodes, it would indicate a biologically significant high functional redundancy in the early stage, and low at the later stage (Wu et al., 2010; Shi et al., 2020). This phenomenon was due to that a large number of nodes removed at the end of the network would result in a sharp reduction in alternative pathways. To analyze the relationship between taxonomic composition within functional groups and abundance of functional genes at species level, the procrustes analysis was performed using the “procrusts” and “protest” functions of the “vegan” R package^11^.

## 3. Results

**Figure 1:**
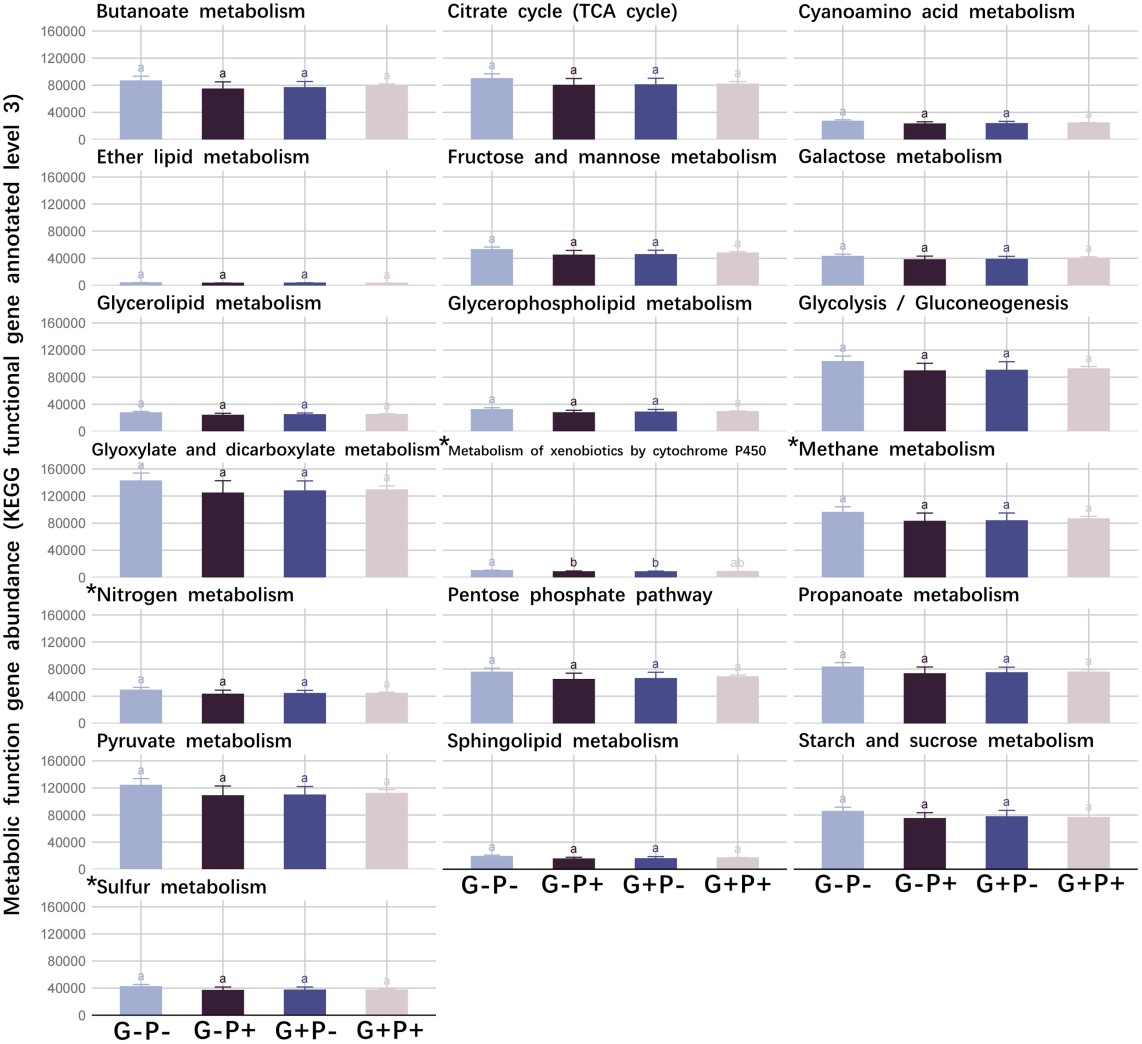
The abundance of functional genes of 15 broad metabolic functions and four specialized metabolic functions under four grazing by P-addition treatments. Means and standard errors are shown. Tukey’s honestly significant difference test was performed under analysis of variance (ANOVAs), with different lowercase letters referring to significant differences between treatments (value of *p* < 0.05). ^*^Represents specialized metabolic functions.

No significant difference was detected in the absolute abundance of all functional genes with broad and specialized metabolic functions among the 4 grazing and P addition treatments (*p* > 0.05), except Metabolism of xenobiotics by cytochrome P450 between G–P– and G + P– or G–P+ (*p* < 0.05), which were significantly decreased by grazing or P addition (Figure 1).

The majority of metabolic pathways of the soil microbial communities were shared among the four grazing by P-addition treatments (Supplementary Figure S1). It was clear that not only N- (specialized) or P-related (broad) metabolic functions, but also the essentially all other metabolic function-related pathways are shared (Supplementary Figure S1).

**Figure 2:**
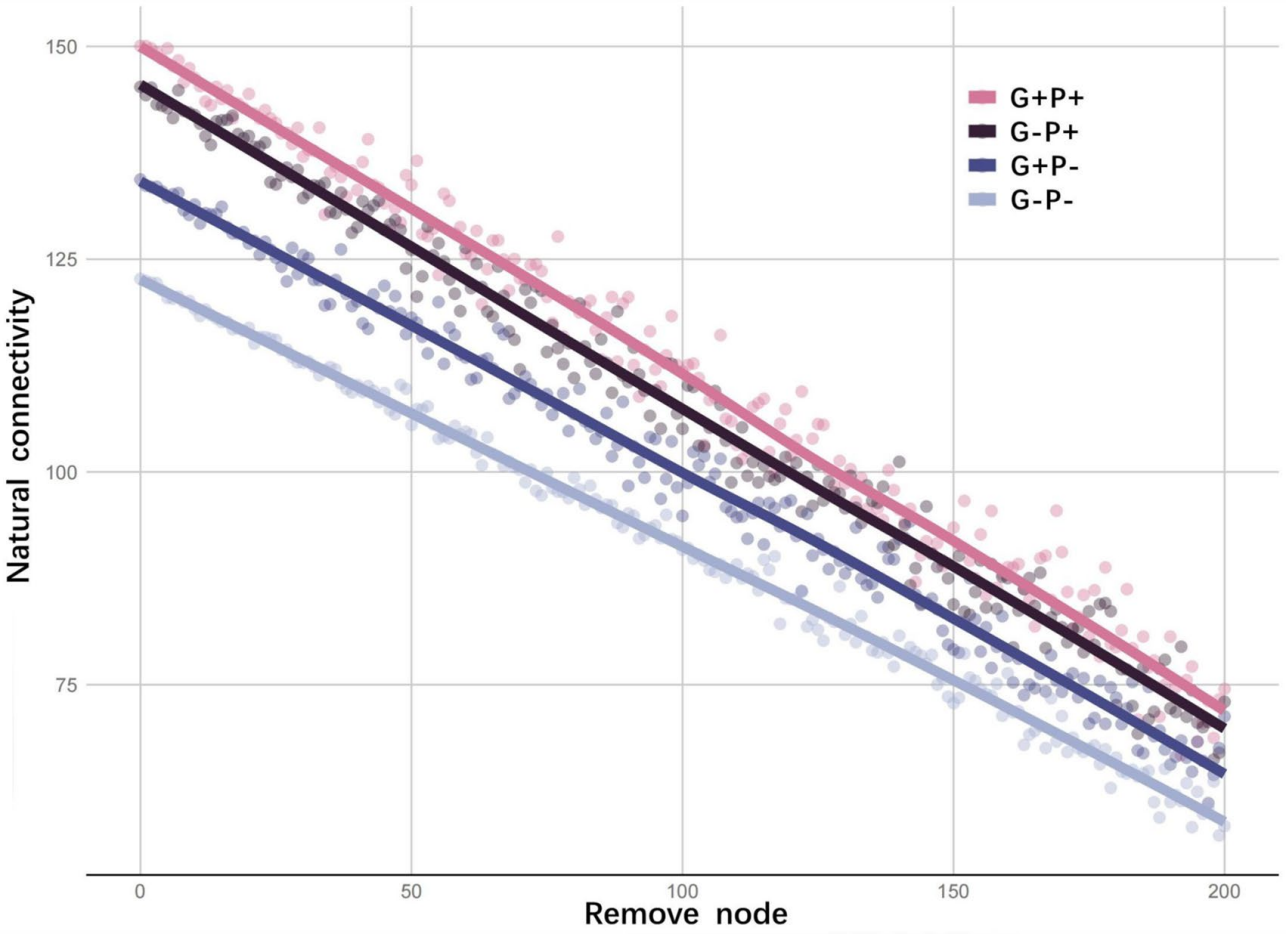
Invulnerability of networks with specialized metabolic functions under four treatments. The natural connectivity of each node is calculated by the adjacency matrix of the specialized functional network. Then 0–200 nodes are removed randomly. The redundancy of alternative pathways in the network is judged by the change trend of natural connectivity. Each point (node) represents a single gene in a specialized metabolic function, the *y*-axis represents the size of the network’s natural connectivity after the random removal of a given number of nodes, and the line represents the fitting of the trend of natural connectivity. The natural connectivity with no nodes removed indicates the network stability of the microbial community.

**Figure 3:**
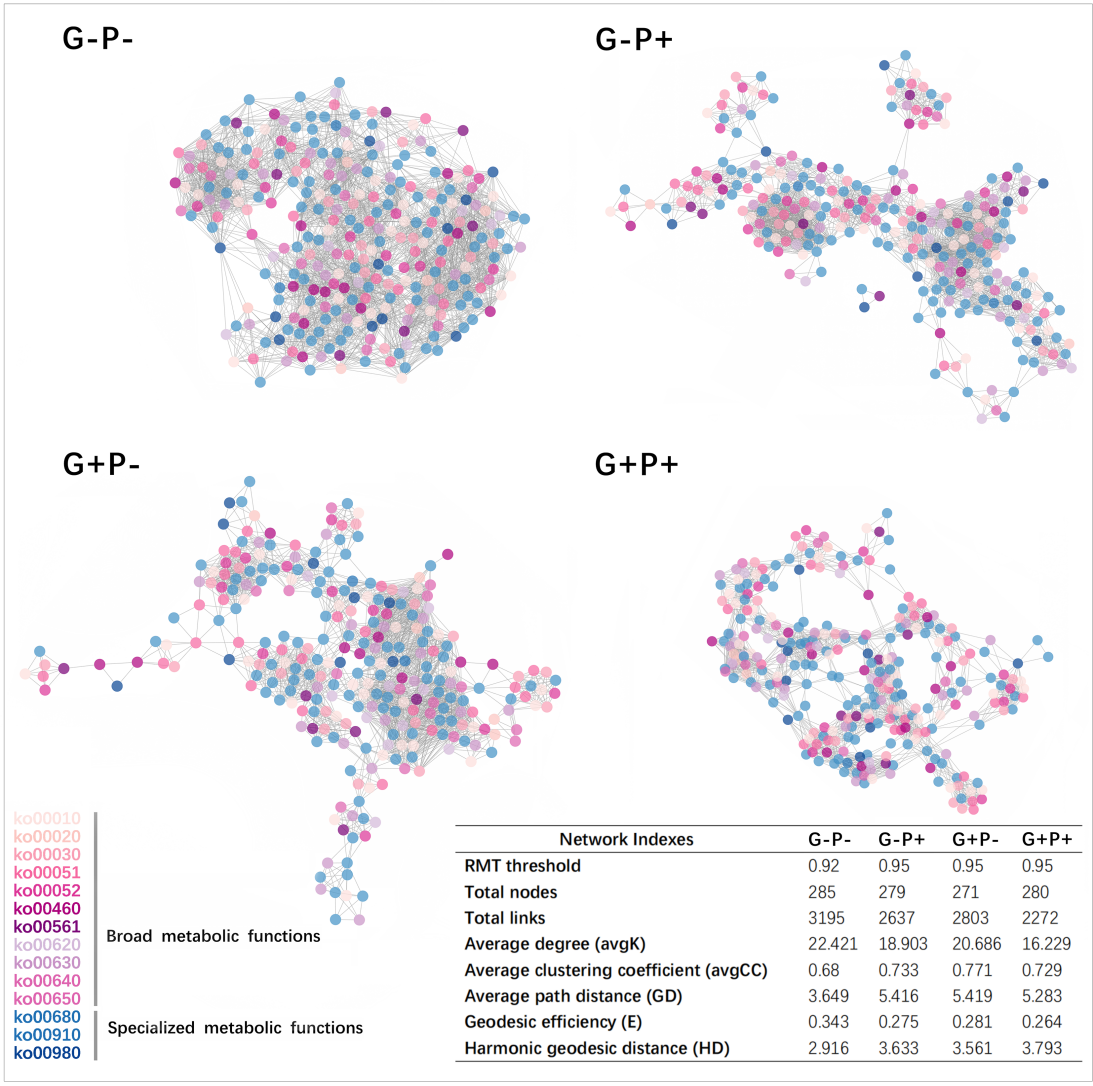
Functional gene co-occurrence networks of soil microbial communities under four grazing by P-addition treatments in a steppe grassland. For a concise visualization, only the 300 most abundant genes in each sample were selected. Five metabolic functions were excluded because of their low abundance. Nodes represent individual genes in the three specialized metabolic functions and 11 broad metabolic functions. The colors of the nodes are the same as that of the Pathway ID shown in legend at the bottom left (Supplementary Table S1), and the table at the bottom right shows important network indexes.

Considering that the presence/absence of a specialized metabolic function alone may not be an accurate measure of functional redundancy in a microbial community, we further explored functional redundancy through ecological networks of microbial communities. From the invulnerability analysis of the specialized metabolic function network, it could be seen that as the number of randomly removed nodes increased, the natural connectivity, as an indicator of the stability of a functional network, decreased linearly in all treatments, may indicating no functional redundancy in the specialized metabolic functions under the four treatments (Figure 2). Moreover, the initial degree of natural connectivity of microbial community (i.e., when no node was removed) differed among treatments (Figure 2). For example, the stability of the specialized functional network of the microbial community was the highest under G + P+ treatment (natural connectivity: 150.06), but lowest under G–P– treatment (134.36). Since broad functions were shared by a large number of species, and the same gene might participate in multiple metabolic cycles, the invulnerability analysis of the network cannot be performed. However, specialized and broad functions were tightly connected in the interaction network, and the effects of specialized and broad functions on the stability of microbial community were similar qualitatively (Figure 3).

The average connectivity (avgK) of the microbial community functional network was the highest under G–P– (22.421) and lowest under G + P+ (16.229). The average clustering coefficient (avgCC) of the network was the lowest under G–P– (avgCC: 0.68) and the highest under G + P– (0.771). Both average path distance (GD) and harmonic geodesic distance (HD) of the network were the smallest under G–P– (3.649), far smaller than under other 3 treatments. In contrast GD and HD, the geodesic efficiency (E) of the network was the largest under G–P– (0.343). Based on all the indices and total number of links, the network under G–P– was the most complex and cohesive, but had the lowest degree of modular structure, while G + P+ had the least number of links, and the lowest E and the highest HD (Figure 3). This result was consistent with the stability of the network of specific metabolic functions in Figure 2, that is, G + P+ was the most stable and G–P– the most unstable community (Figure 2).

**Figure 4:**
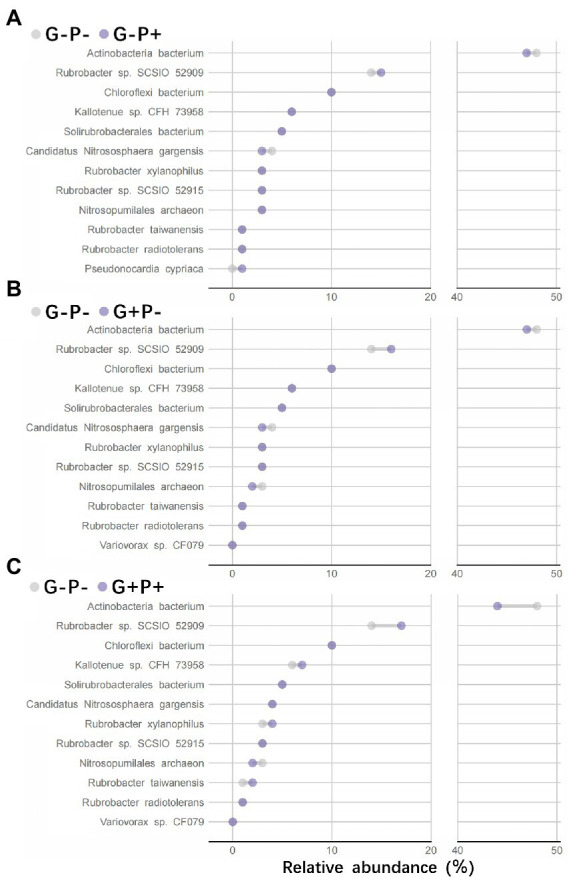
The variation trend of species relative abundance within functional groups between no-grazing and no-P addition control (G–P–) and other three treatments of P addition only (**A**: G–P+), grazing only (**B**: G + P–), and both P addition and grazing (**C**: G + P+). The top 12 species with the highest relative abundance in the functional groups (more than 95% of the total species abundance) were selected, and their metabolic functional gene abundance was also the highest both in broad and specialized functions.

**Figure 5:**
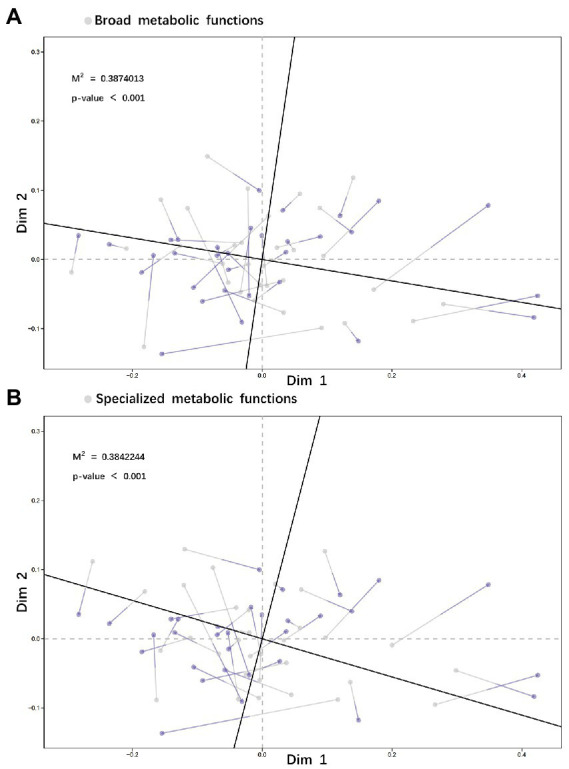
Species within functional groups based on the NMDS (Bray–Curtis) were correlated with broad metabolic functions **(A)** and specialized metabolic functions **(B)** in a procrustes analysis (Permutations = 999). The 20 species with the highest relative abundance in the functional groups (more than 95% of the total species abundance) were selected, and their metabolic functional gene abundance was also the highest both in broad and specialized functions.

Comparison of species relative abundance within functional groups between no-grazing and no P-addition control (G–P–) and other three treatments showed that (i) adding P only (G–P+) or grazing only (G + P–) or both adding P and grazing (G + P+) decreased the relative abundance of species *Actinobacteria bacterium*, but increased that of species *Rubrobacter* sp. *SCSIO 52909*, and the increase or decrease was the most striking by G + P+ treatment, while the other two treatments were relatively weak (Figures 4A–C). On the basis of Non-metric Multidimensional Scaling (NMDS) analysis of both species relative abundance and broad or specialized metabolic functions within functional groups, a procrustes analysis of species within functional groups and broad/specialized metabolic functions showed a significant correlation between microbial community composition within functional groups and function in different samples (broad metabolic functions: *M*^2^ = 0.387, value of *p* <0.001, Figure 5A; specialized metabolic functions: *M*^2^ = 0.384, value of *p* < 0.001, Figure 5B). Moreover, the correlation (*M*^2^) between broad and specialized functions and species composition within functional groups was almost identical.

## 4. Discussion

Our results confirm that the abundance of functional genes of free-living microbial communities in grassland soils remains unchanged under grazing and P addition by metagenomic approach, consistent with the results observed in other studies on different environmental gradients or in different habitats (Fernandez-Gonzalez et al., 2016; Louca et al., 2016a,b; Song et al., 2022). However, we found that the variation of taxonomic composition and functional composition is not decoupled, but has a strong consistency when noise is eliminated (taxonomic species composition within functional groups).

### 4.1. Grazing and P addition reduced complexity and cohesion of functional gene co-occurrence networks

Scholars have no an agreed definition of stability (Paine, 1969; Coyte et al., 2015; Butler and O’Dwyer, 2018) and functional redundancy. For example, Fuhrman et al. (2015) have argued that only when organisms are easily interchangeable due to a high degree of ecological similarity, their function is redundant; Allison and Martiny (2008) define functional redundancy as an organism that can perform a function of another organism at the same rate, and considering the realizability of the actual operation (Fuhrman et al., 2015), the number of alternative pathways is still used as a measure of functional redundancy. This is an indirect measurement that determines the existence of functional redundancy by whether the reduction of alternative pathways causes the stability of a functional network, i.e., the capacity of a functional network to withstand random failures, to show a nonlinear trend (Wu et al., 2010; Shi et al., 2020). This is based on the positive relationship between functional redundancy and ecological stability (Biggs et al., 2020), and on the premise that functions with more species have higher stability (Díaz and Cabido, 2001). However, our results do not show an obvious functional redundancy of functional network in the studied system.

Although reductions in the complexity of functional gene networks have been observed in other nutrient addition experiments (Ma et al., 2022), co-occurrence networks with correlations can only partially explain the phenomenon and cannot clearly explain why (Deng et al., 2012; Ma et al., 2022). In our results, networks built on individual gene do interact less with nutrient addition than with no change in overall metabolic functions. From the niche perspective, we hypothesize that nutrient addition reduces the niche dimension (both P addition and grazing are one of the niche dimensions) (Harpole and Tilman, 2007). This may lead to a reduction in gene interactions due to environmental filtering (Li et al., 2019). However, microbial nutrient strategy-related traits are highly conserved, and overall metabolic function may not change significantly due to changes in individual gene (Martiny et al., 2015). This may also imply that life history strategies, such as high growth rates, of a few dominant taxa can explain more variation than functional traits. This does not mean that the stability of functional gene co-occurrence networks and noise species are unimportant. At any point in time, ~80% of microbial cells are dormant (Blagodatskaya and Kuzyakov, 2013), and although the effect of nutrient addition on microbial cell dormancy is unclear, the impact on ecosystems is huge just considering the presence of a large number of microbial necromass (Sokol et al., 2022). For example, it has been shown that the addition of N can increase the proportion of dormant microbial taxa in salt marshes from 45 to 90%; and at the same time, there is no significant change in the overall microbial community, suggesting that relative stability in overall diversity or abundance may not be an efficient indicator for dramatic changes in microbial communities (Kearns et al., 2016). In addition, microbes usually control compensatory functions (e.g., division of labor and metabolic exchange), which may make redundancy less easily observed (West and Cooper, 2016; Cooper et al., 2022).

### 4.2. “Coupling” of microbial community function and taxonomy dominated by very few microorganisms

Our study shows that the relative abundance of species *Actinobacteria bacterium* (species A) and *Rubrobacter* sp. *SCSIO 52909* (species R) is dominant and complementary in the soil microbial community in response to grazing or P addition. The relative abundance of other species varied little, with a variation range less than 1%. Species A and R were still dominant (together they accounted for about 50% of the relative abundance), although the complementation between control and grazing alone or P-addition alone was not obvious.

The treatment such as grazing or P addition alters cycling of soil nutrients, for example, C, N, or P. In plant ecology, Tilman’s classical competition theory states that in steady-state and well-mixed systems, at most, any given resource can limit only one continuous population. This population will be one that is able to maintain a stable size at the lowest possible resource level, since all other populations will either win out in competition or be limited by different resources (Tilman, 1982). This theory is more instructive in microbial ecology than in macroecology, because in large plants and animals, spatial and temporal heterogeneity of environmental conditions coupled with differences in response between species can effectively create multiple niches, making populations with extreme traits difficult to observe (Fierer et al., 2014; Louca et al., 2018). Taking into account the very clear coexistence of various microbial communities, as species with extreme functional abundance and wide metabolic versatility in their functional spaces (Mouillot et al., 2013; Van Bergeijk et al., 2020), species A (Only resource limited) and R (uniquely complementary) win the competition even though different treatments limit different minimum resource levels, for example, if a treatment limits N, then the species with the lowest demand for N will gain a competitive advantage. The functional changes were “coupled” with the changes in species abundance, which may be the reason why the relative abundance and the functional gene abundance complementary simultaneously were observed in different treatments. The complementarity of functional gene abundance leads to the constant functional composition, which ultimately results in the “decoupling” of taxonomic and functional composition in the microbiome. In addition, two most abundant species dominated the metabolic function of the community and have stable functions, but no functional redundancy, which is consistent with our conclusion. This is also consistent with the conclusion that functional redundancy is not related to species richness *per se* (Biggs et al., 2020). In general, these conclusions are consistent with the view that ecological redundancy is derived from functional compensation (Walker, 1992), but functional compensation between only two species is not sufficient to support the emergence of functional redundancy for large microbial richness.

### 4.3. Bistability mechanism leads to coexistence of two species

As for the competition between species A and R, although A is the only species restricted to reduce relative abundance, R is also the only one to fill the gap. Tilman’s classical competition theory does not explain why there are two and only two competition winners in soil microbial communities, even though it is modified to guide bacterial growth under a single restricted resource (Monod, 1949; Tilman, 1977, 1985). Although imaginative scholars have been characterized by the division of labor effect (Giri et al., 2019; Oña and Kost, 2022) or stable marriage problem (Goyal et al., 2018) to explore the competition and coexistence between microorganisms, but the competition between microorganisms may not be as complex as commonly thought.

Antibiotics are important agents of competition among microorganisms, and the ecological purpose of the competition is to gain advantage in resource acquisition (Van Bergeijk et al., 2020). Some studies suggest that antibiotics really act as intraspecific and interspecific signaling molecules (Hibbing et al., 2009), which is extremely rare in large organisms (Mouillot et al., 2013). Based on the inhibitory effect of antibiotics, Wright & Vetsigian argue that competition among microorganisms is not the survival of the fittest based on a competitive exclusion principle, nor is it based on a circular dominance network based on intransitive competition (similar to a rock-scissors-paper game), but on a bistability mechanism (Kassen and Rainey, 2004; Reichenbach et al., 2007; Wright and Vetsigian, 2016; Levine et al., 2017). In simple terms, the bistability mechanism refers to the inter-strain inhibition of *Streptomyces* in soil samples that favors the high abundance of strains to resist the invasion of other strains. At the same time, strains are also unable to invade other strains at low abundance, and coexisting groups of three or more inhibitory strains may exhibit frequent paired bistability and ultimately result in multiple rather than single winners in the microbial community (Kelsic et al., 2015; Wright and Vetsigian, 2016). This mechanism is consistent with our observations.

First, both grazing and P addition in our study lead to increased one or more soil nutrient cycling, which is supported by the results of Guitian and Bardgett (2000), Kohler et al. (2005), and Dai et al. (2019). This means that the soil enters a relatively nutrient-rich state, and nutrient-rich conditions hinder the growth of *Actinomycetes* and the production of antibiotics (Rigali et al., 2008; Urem et al., 2016; Lee et al., 2018). This narrows the “bacteriostatic circle” of species A and is filled by slow-growing species R (Suzuki, 2015), which is extremely resistant to radiation (Suzuki, 2015), high temperature (Chen, 2004), and even drought (Ferreira et al., 1999). This is consistent with the conjecture for the distribution of mosaics under resource overlap (Wright and Vetsigian, 2016).

Secondly, most of the top 20 species identified in functional groups belong to *Actinobacteria*, even *Streptomyces* and *Rubrobacter*. Although only one or two strains of each species of the genus *Rubrobacter* are described and intra-species variability is unknown, they share many common phenotypic and chemical taxonomic characteristics (Chen, 2004; Suzuki, 2015). This raises the question why only species R is able to fill vacancies among multiple species with similar characteristics? The answer to this question may come from feature of bistability mechanisms, namely that bistability phenomena prevalent among species have a strong influence on community assembly that is beyond the expectations of models based on environmental filtration and diffusion (Powell and Bennett, 2016; Wright and Vetsigian, 2016). This effect on community assembly stems from the extreme sensitivity of bistability mechanisms to the initial abundance of species arriving at the same time, because species with higher abundance are better able to resist invasion, an advantage of abundance itself (Wright and Vetsigian, 2016). Therefore, the fact that only species R can fill the gap may be resulted from its higher abundance at the beginning of community establishment, rather than some unknown advantage of species R.

### 4.4. Ecosystem functions are independent of soil nutrient changes

Both species A and R have a complete functional classification, and the functional and taxonomic composition are consistent, which is the reason for the constant metabolic function. In addition to competition, bacteria, especially high-abundant bacteria, also participate in multi-cellular level behaviors that require cooperation, such as quorum sensing (Hense et al., 2007). High-abundance inhibitory species can actually benefit themselves by investing in common products, such as extracellular proteases and siderophores (West and Buckling, 2003; West et al., 2007; Allen et al., 2013). Therefore, the contribution of species A and R to soil metabolic function is beneficial to other species, but it still mainly brings advantages to themselves. Other low-abundant species that also have complete functional classifications may also become high-abundant species at different times and spaces due to different life cycles and different spatial distributions (Moll and Brown, 2008; Eldridge et al., 2015). This is not contrary to the rule that Actinobacteria, as the representative of K strategy, live in an oligotrophic environment and sacrifice growth rate to maximize resource acquisition (Zhou et al., 2017).

In summary, our study demonstrates that grazing and P addition only affect the complementarity of relative abundance and functional composition of the two dominant species, this may indicate that metabolic function is not related to nutrient limitation, but to inhibition between dominant species. In fact, no evidence has been reported for differences between qualitative predictors of soil nutrient limitation, these include distance, burning, grazing, tillage, and soil texture (Fay et al., 2015), so the high abundance and bistability mechanisms of Actinobacteria in soils may be widespread and metabolic functions independent of nutrient content may be universal. Future global-scale metagenomic studies may provide more definitive evidence to prove or disprove this prediction.

## 5. Conclusion

Our study demonstrates that species and function of microorganisms are not decoupled in terms of taxonomic species composition within functional groups. On the contrary, the close coupling and complementarity of relative abundance and functional composition between dominant species resulted in no change in ecosystem function across grazing and P addition treatments. Moreover, the relative abundance and functional composition of dominant species A and R are closely coupled and complementary, resulting in the illusion of functional redundancy. Thus, our results suggest that nutrient change does not affect ecosystem function, whereas inter-species inhibition does.

Our conclusion has some limitations, such as no significant changes are detected in the dominant microbial species across the experimental treatments. This may be related with the small scale of the experimental plots, which may lead to the migration of dominant species to cover the whole site. This may create uncertainty about the prevalence of bistability mechanisms in the large scale. Moreover, we cannot measure the antibiotic secretion of dominant species in the field, and this direct evidence may be difficult to achieve, given the presence of “cryptic” gene clusters (Van Bergeijk et al., 2020). Also, due to the large errors inherent in field data, we still cannot rule out that the phenomenon observed in this study may simply be facilitated by a decrease in the abundance of the species with the largest relative abundance. However, our study may change stereotypes about the impact of nutrient changes on nutrient cycling in ecosystems and highlight the need to strengthen further research on interactions between very few important microorganisms. In addition, the abundance of functional genes or enzyme activities in microbial communities varies on environmental gradients, including the gradients of nutrient input quantity or grazing intensity (Guo et al., 2015; Leff et al., 2015; Liu et al., 2023). However, our study does not involve the effects of different levels of treatments, as microbial communities in bulk soil of the studied ecosystem change little compared to that in rhizosphere (Liu et al., 2023). The intensity of the P addition and animal grazing treatment is high in this study, thus the results may mean that neither intense P addition nor grazing cannot lead to a pronounced change in metabolic function. More attention should be paid to the effects of environmental gradients on microbial communities and subsequent changes in ecosystem functions.

## Data availability statement

The datasets presented in this study can be found in online repositories. The names of the repository/repositories and accession number(s) can be found in the article/Supplementary material.

## Author contributions

FL and JL: conceptualization and study design. JL, CS, YL, KT, QF, QY, XG, NW, and HL: field and laboratory experiments. HL: data analysis and manuscript writing. WG: manuscript reviewing. All authors contributed to the article and approved the submitted version.

## Funding

This research was supported by Department of Science and Technology of Inner Mongolia Autonomous Region of China (Grant Nos. 201503001 and 2019007) and by National Natural Science Foundation of China (Grant No. 32071564).

## Conflict of interest

The authors declare that the research was conducted in the absence of any commercial or financial relationships that could be construed as a potential conflict of interest.

## Publisher’s note

All claims expressed in this article are solely those of the authors and do not necessarily represent those of their affiliated organizations, or those of the publisher, the editors and the reviewers. Any product that may be evaluated in this article, or claim that may be made by its manufacturer, is not guaranteed or endorsed by the publisher.

## Footnotes

^1^ www.illumina.com

^2^ https://github.com/OpenGene/fastp

^3^ https://github.com/voutcn/megahit

^4^ http://metagene.cb.k.u-tokyo.ac.jp/

^5^ http://www.bioinformatics.org/cd-hit/

^6^ http://soap.genomics.org.cn/

^7^ http://www.diamondsearch.org/index.php

^8^ http://www.genome.jp/kegg/

^9^ https://pathways.embl.de/

^10^ http://ieg4.rccc.ou.edu/MENA/

^11^ https://github.com/vegandevs/vegan
