# Supplementary material for: The reciprocal changes in dominant species with complete metabolic functions explain the decoupling phenomenon of microbial taxonomic and functional composition in a grassland
Source: Front Microbiol. 2023 Mar 16;14:1113157. doi: 10.3389/fmicb.2023.1113157 (PMC10060659; doi:10.3389/fmicb.2023.1113157)
Supplement: Supplementary file 1 [file Data_Sheet_1.docx]

Supplementary Material

The reciprocal changes in dominant species with complete metabolic functions explain the decoupling phenomenon of microbial taxonomic and functional composition in a grassland

Huaiqiang Liu, Frank Yonghong Li*, Jiayue Liu, Chunjun Shi, Kuanyan Tang, Qianhui Yang, Yu Liu, Qiang Fu, Xiaotian Gao, Ning Wang, Wei Guo

*** Correspondence:** Frank Yonghong Li: lifyhong@126.com

**Supplementary Table 1.** The classification of broad and specialized metabolic functions based on the KEGG database.

| **Specialized metabolic functions** | | | | |
| --- | --- | --- | --- | --- |
| No. | Level 1 | Level 2 | Level 3 | Pathway ID |
| 1 | Metabolism | Energy metabolism | Methane metabolism | ko00680 |
| 2 | Metabolism | Energy metabolism | Nitrogen metabolism | ko00910 |
| 3 | Metabolism | Energy metabolism | Sulfur metabolism | ko00920 |
| 4 | Metabolism | Xenobiotics biodegradation and metabolism | Metabolism of xenobiotics by cytochrome P450 | ko00980 |
| **Broad metabolic functions** | | | | |
| No. | Level 1 | Level 2 | Level 3 | Pathway ID |
| 1 | Metabolism | Carbohydrate metabolism | Glycolysis / Gluconeogenesis | ko00010 |
| 2 | Metabolism | Carbohydrate metabolism | Citrate cycle (TCA cycle) | ko00020 |
| 3 | Metabolism | Carbohydrate metabolism | Pentose phosphate pathway | ko00030 |
| 4 | Metabolism | Carbohydrate metabolism | Fructose and mannose metabolism | ko00051 |
| 5 | Metabolism | Carbohydrate metabolism | Galactose metabolism | ko00052 |
| 6 | Metabolism | Carbohydrate metabolism | Starch and sucrose metabolism | ko00500 |
| 7 | Metabolism | Carbohydrate metabolism | Pyruvate metabolism | ko00620 |
| 8 | Metabolism | Carbohydrate metabolism | Glyoxylate and dicarboxylate metabolism | ko00630 |
| 9 | Metabolism | Carbohydrate metabolism | Propanoate metabolism | ko00640 |
| 10 | Metabolism | Carbohydrate metabolism | Butanoate metabolism | ko00650 |
| 11 | Metabolism | Metabolism of other amino acids | Cyanoamino acid metabolism | ko00460 |
| 12 | Metabolism | Lipid metabolism | Ether lipid metabolism | ko00565 |
| 13 | Metabolism | Lipid metabolism | Glycerolipid metabolism | ko00561 |
| 14 | Metabolism | Lipid metabolism | Glycerophospholipid metabolism | ko00564 |
| 15 | Metabolism | Lipid metabolism | Sphingolipid metabolism | ko00600 |

**Supplementary Figure 1.** KEGG metabolic pathway map of the soil microbial communities under four grazing (G±) and P-addition (P±) treatments in a steppe grassland. The dark grey pathways or enzymes are shared by all treatments, while the grayish blue, dark moderate blue, dark magenta and pink pathways or enzymes are occupied only by G–P–, G+P–, G–P+ or G+P+ treatment, respectively. The metabolisms represented by the other colors in the figure are the default iPath map information (Darzi et al., 2018).


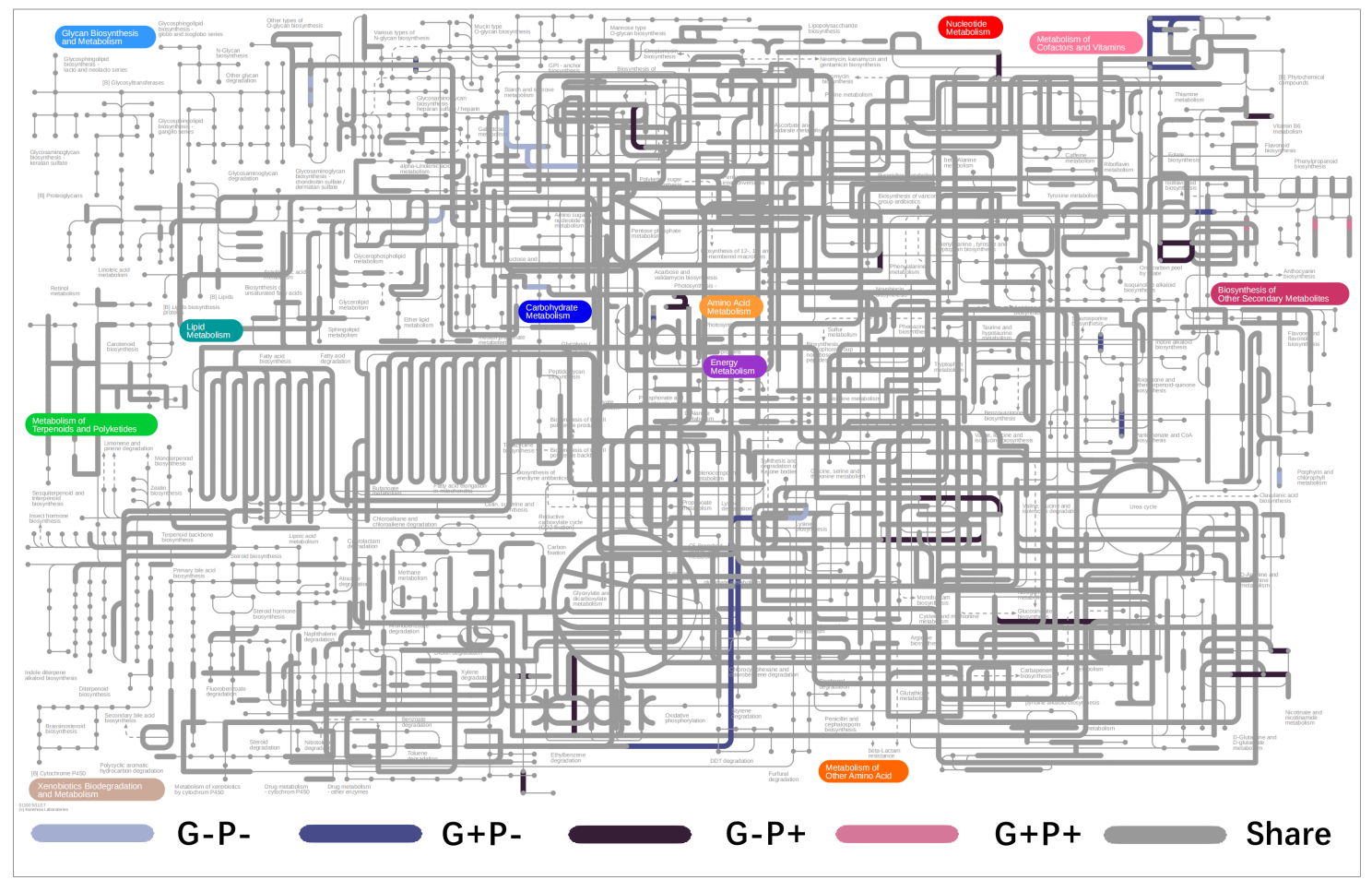


Darzi, Y., Letunic, I., Bork, P., & Yamada, T. (2018). iPath3.0: interactive pathways explorer v3. *Nucleic Acids Res.* 46(W1), W510–W513. doi:10.1093/nar/gky299
